# Exploring the Therapeutic Potential of Membrane Transport Proteins: Focus on Cancer and Chemoresistance

**DOI:** 10.3390/cancers12061624

**Published:** 2020-06-19

**Authors:** Shekoufeh Almasi, Yassine El Hiani

**Affiliations:** 1 Department of Cellular and Molecular Medicine, Faculty of Medicine, University of Ottawa, Ottawa, ON KIH 8M5, Canada; salmasi@uottawa.ca; 2 Department of Physiology and Biophysics, Faculty of Medicine, Dalhousie University, Halifax, NS B3H 4R2, Canada

**Keywords:** membrane transporters, pumps, ion channels, cancers, chemoresistance

## Abstract

Improving the therapeutic efficacy of conventional anticancer drugs represents the best hope for cancer treatment. However, the shortage of druggable targets and the increasing development of anticancer drug resistance remain significant problems. Recently, membrane transport proteins have emerged as novel therapeutic targets for cancer treatment. These proteins are essential for a plethora of cell functions ranging from cell homeostasis to clinical drug toxicity. Furthermore, their association with carcinogenesis and chemoresistance has opened new vistas for pharmacology-based cancer research. This review provides a comprehensive update of our current knowledge on the functional expression profile of membrane transport proteins in cancer and chemoresistant tumours that may form the basis for new cancer treatment strategies.

## 1. Introduction

In mammalian cells, the plasma membrane is a selectively permeable barrier that creates an intracellular environment and maintains cell stability and homeostasis. The proper functioning of the plasma membrane is dependent on a group of membrane transport proteins that permit the selective transport of essential substances for the survival and development of the organism [1]. To date, three different types of membrane transport proteins have been described: (1) ATP-powered pumps or ATPases which actively transport solutes against their electrochemical gradients; (2) channel proteins which facilitate the passive diffusion of ions following their electrochemical gradients; and (3) facilitators which move solutes either up or down their gradients. When the gates of the transporters are open, the selective flux of metabolites and ions occurs that affects a wide range of cellular processes such as membrane potential (due to the ion exchange), cell volume (due to the water permeation coupled to ion transport), and cell signaling (due to the impact on the function of ions/metabolites or intracellular effectors). All of these events are critical in determining cell fate to survival, death, or malignant transformation [2]. Another important role of membrane transport proteins is to maintain a balance between toxicity and effectiveness of chemotherapeutics by controlling drug uptake, disposition, and clearance [3,4,5,6]. Therefore, disturbance in the expression profile of membrane transport proteins is often associated with tumourigenesis and/or chemoresistance [7,8]. In this review, we will discuss the correlations between membrane transporters (pumps and channels) and cancer progression as well as chemoresistance (Appendix A).

## 2. Membrane Pumps

**Figure 1:**
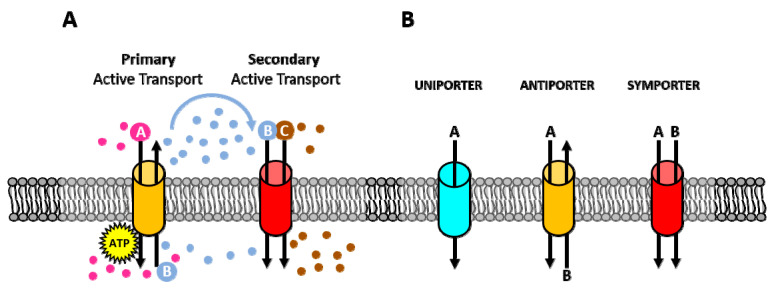
Different types of ion transport. (**A**) Active and secondary transport: Primary active transporter uses ATP to move ions across the membrane [A and B], against their electrochemical gradients to create an electrochemical gradient. Secondary active transporter uses the electrochemical gradient generated by primary active transporters to move one molecule down its gradient [B] while transporting another molecule against its electrochemical gradient [C]. (**B**) Uniporter, antiporter, and symporter: Uniporter carries one molecule or ion in one direction. Antiporter carries two different molecules or ions in opposite directions. Symporter also carries two different molecules or ions in the same direction.

Membrane pumps are transmembrane proteins that facilitate the active transport of various substances against their electrochemical gradients. Mechanistically, membrane pumps can be divided into two main categories: primary and secondary active transporters. Through ATP hydrolysis, primary active transporters move solutes against their electrochemical gradients. These pumps are often uniporters which are involved in the active transport of a single molecule across the cell membrane. Instead, secondary active transporters utilize the energy stored in the electrochemical gradient of ions across the plasma membrane that was generated by the primary active transporters. Therefore, in this type of transport, the transfer of one molecule down its gradient is coupled to the movement of another molecule against its gradient (Figure 1A). Depending on the direction of transport, two types of secondary active transporters have been described: antiport pumps that transport two molecules in opposite directions and symport pumps that move both molecules in the same direction (Figure 1B) [9].

The crucial role of membrane pumps in conducting the active transport of a wide range of substrates including ions, amino acids, large polypeptides, and essential metabolites highlights their indispensable function in maintaining cellular homeostasis [10]. Moreover, membrane pumps are also involved in drug uptake and efflux that impact disposition and cytotoxic effects of anticancer drugs [11,12]. In this context, membrane transporters can act as importers and mediate the transport of drugs into the cell or function as exporters and pump substances outside the cell. In cancer, altered expression of membrane pumps often correlates with chemoresistance (Appendix A) [13,14,15]. The following sections will highlight the relationship between membrane pumps and cancer progression as well as chemoresistance.

### 2.1. Na^+^/K^+^-ATPase

**Figure 2:**
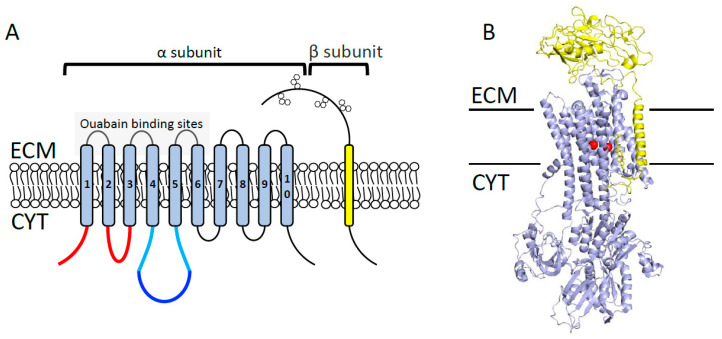
Na^+^-, K^+^-ATPase overall structure. (**A**) Na^+^-, K^+^-ATPase consists of a catalytic α subunit and a regulatory β subunit. The α subunit consists of 10 transmembrane helices, harboring 3 different cytoplasmic domains: the actuator responsible for dephosphorylation (shown in red); the nucleotide-binding, responsible for ATP binding (shown in blue); and the phosphorylation domains (shown in cyan). The β subunit consists of one transmembrane helix with a large glycosylated extracellular domain (shown in hexagon orange boxes). ECM = extracellular milieu; CYT = cytoplasm. (**B**) Overall domain architecture of Na^+^/K^+^ transporter in the Na^+^-bound state (Protein Data Bank [PDB] code 4HQJ). Catalytic α subunit is colored in blue, β subunit is shown in yellow, and Na^+^ ions are shown in red.

The plasma membrane sodium pump (Na^+^/K^+^-ATPase) is a hetero-dimeric complex that consists of catalytic a- and regulatory b-subunits (Figure 2). Four different isoforms of a-subunit and three isoforms of b-subunit exist in human cells [16,17,18]. Functionally, Na^+^/K^+^-ATPase is a ubiquitous P-type ATPase transporter that exchanges three Na^+^ for two K^+^, thus establishing plasma membrane potential. The generated membrane potential is further required for accelerating the central cellular processes including secondary active transport of metabolites and cell excitability [19,20]. Na^+^/K^+^-ATPase is naturally activated and deactivated by ATP and cardiotonic steroids (e.g., ouabain, digitoxin), respectively [21,22]. Over the last decades, an association between Na^+^/K^+^-ATPase and etiology of several malignancies, including breast, non-small cell lung cancer, glioblastoma, and melanoma has been established [23,24]. For instance, the expression level of a-subunit (isoforms 1 and 3) is increased in various cancers, therefore its pharmacological inhibition has been proposed to improve cancer therapy [25,26,27]. Specifically, several studies demonstrated that the a1-subunit of Na^+^/K^+^-ATPase is highly expressed in glioblastomas and that its inhibition decreases cell proliferation and migration while increasing survival of the orthotopic patient-derived xenograft mouse model of human glioblastoma [28,29,30,31]. Similarly, pharmacological inhibition of a1-subunit reduces tumour progression and induces apoptosis in prostate cancer [32] and lung cancer cells [33]. Furthermore, Rajasekaran et al. reported that the expression level of β1-subunit is markedly reduced in renal cell carcinoma and that its ectopic expression inhibits the invasiveness and motility of these cells. For the mechanism, authors showed that elevated Na^+^/K^+^-ATPase positively impacts E-cadherin-mediated formation of tight junctions and epithelial cell polarity [34,35]. Likewise, reduced β_1_-subunit correlates with poorly differentiated breast (MDA435), colon (SW480), pancreas (MiaPaCa-2), and kidney (MSV-MDCK) cancer cells. This evidence suggests that the Na^+^/K^+^-ATPase β1-subunit plays a tumour-suppressor role in cancer [36,37].

In addition to its significance in cell proliferation and invasion, Na^+^/K^+^-ATPase is also emerging as an effective therapeutic target to overcome chemoresistance [38]. The first line of evidence was reported in 1991 when Andrews et al. showed that the pharmacological inhibition of Na^+^/K^+^-ATPase with ouabain reduced the intracellular accumulation of cisplatin by 50% in a 2008 ovarian carcinoma cell line [39], suggesting that low Na^+^/K^+^-ATPase is linked to cisplatin resistance. Further investigations demonstrated that inhibition of Na^+^/K^+^-ATPase expression and function promotes cisplatin-resistance in leukemia [40], non-small lung cancer [41,42], and prostate cancer cell lines [43]. For example, Na^+^/K^+^-ATPase is significantly reduced in cisplatin-resistant BHY oral squamous cell carcinoma cells, and its further inhibition exacerbates the cisplatin-resistant phenotype [44]. Similarly, Na^+^/K^+^-ATPase is considerably reduced in oxaliplatin-resistant ovarian carcinoma cells C10B, therefore its ectopic expression enhances oxaliplatin accumulation and promotes oxaliplatin-mediated cell death [45]. Together, these findings reveal that Na^+^/K^+^-ATPase may serve as a potential therapeutic target for the treatment of chemoresistant malignancies. However, further investigations are required for the development of a novel and high-affinity molecule to target Na^+^/K^+^-ATPase for cancer treatment.

### 2.2. SERCA

**Figure 3:**
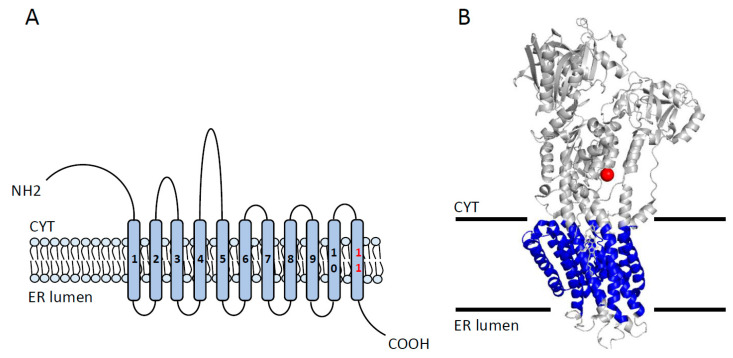
The overall structure of SERCA (Sarco/endoplasmic reticulum Ca^2+^ ATPase) pump. (**A**) The topology of SERCA showing 10 transmembrane segments (TMS (transmembrane segment) 11 is found only in SERCA2b), with a large cytoplasmic N-terminal, large cytoplasmic loops, and a luminal C-terminal. (**B**) Overall 3D-architecture of SERCA transporter in the E2-state complexed with a Thapsigargin derivative Boc-(phi)Tg (Protein Data Bank [PDB] code 3NAN). Cytoplasmic and luminal loops are shown in gray, TMSs are shown in blue, and Tg inhibitor is shown in red. CYT = cytoplasm; ER lumen = endoplasmic reticulum lumen.

The Sarco/endoplasmic reticulum Ca^2+^ ATPase (SERCA) is a P-type ATPase located in the sarcoplasmic reticulum (SR) within myocytes [46]. In humans, three genes encoding three major paralogs of SERCA (SERCA 1, 2, and 3) have been described. In total, 11 different isoforms (SERCA1a–1b, SERCA2a–2c, and SERCA-3a–3f) of SERCA are variably expressed across human cells and tissues [47]. For example, SERCA2b is ubiquitously expressed while the expression of SERCA1a and SERCA 2a is restricted to skeletal and cardiac muscles, respectively [48]. Structurally, SERCA isoforms show a modular architecture consisting of three cytosolic domains responsible for ATP binding and hydrolysis and one transmembrane domain involved in Ca^2+^ binding and transport (Figure 3) [49]. Functionally, SERCA pumps two Ca^2+^ ions from the cytosol into the SR lumen coupled with the hydrolysis of a single ATP, therefore establishing a 1000-fold Ca^2+^ gradient across the SR and cytosolic compartments [50,51].

The proper maintenance of the SR Ca^2+^ gradient is vital in a vast array of cellular functions, such as cell proliferation, invasion, and cell death [52,53,54]. Given the significance of the above functions in cancer development, dysregulated SERCA is associated with various cancers [55]. For instance, Prasad et al. demonstrated that SERCA2 knockout mice are highly susceptible to developing squamous-cell carcinoma, emphasizing the link between impaired SERCA and carcinogenesis [56]. Furthermore, the expression profile of SERCAs is highly diverse in human carcinomas [57,58]. For example, downregulated SERCA2 plays a key role in the progression of lung and thyroid cancers [59,60]. On the contrary, the upregulation of SERCA2 in colorectal carcinoma is correlated with serosal invasion, lymph node metastasis, and advanced tumour stage [61]. Like SERCA2, SERCA3 expression is differentially altered in various cancer types. For instance, SERCA2 levels are reduced in colorectal carcinoma and breast cancers [62,63,64,65], while they increase in myeloid leukemia [66] and gastric cancer [63].

Interestingly, the aberrant expression of SERCAs is also associated with chemoresistance. In this aspect, several studies reported that SERCA1–3 are markedly decreased in cisplatin-resistant MDAH-2774 ovarian cancer cell lines [67], and in low-level cisplatin-resistant non-small-cell lung cancer cells H1339 [68]. A link between altered SERCA and cancer has led to the development of several modulators to either activate/restore or inhibit SERCA for the treatment of different cancers with dysregulated or impaired SERCA. The generated drugs have been used in cancer therapy either individually or in combination with chemotherapeutics [69,70]. Indeed, various inhibitors of SERCA such as thapsigargin, cyclopiazonic acid, and curcumin have been widely used as anticancer drugs in numerous cancers. Furthermore, short-chain fatty acids (e.g., butyrate, valerate, and caproate) and resveratrol have been shown to induce SERCA3 expression and inhibit cell survival in gastrointestinal carcinoma and breast cancer, respectively [63,71]. A curcumin analog F36 is an example of a SERCA inhibitor which has been shown to reduce the proliferation of colorectal cancer cells through inhibiting SERCA2 expression [72]. More recently, the small molecule CXL017 has been demonstrated as a potent anticancer drug in several chemoresistant leukemia cell lines [73,74,75]. However, further studies are required to confirm the effectiveness of CXL017 in other cancers and to test whether CXL017 can promote the effectiveness of conventional chemotherapeutics.

### 2.3. Vacuolar ATPase (V-ATPase)

**Figure 4:**
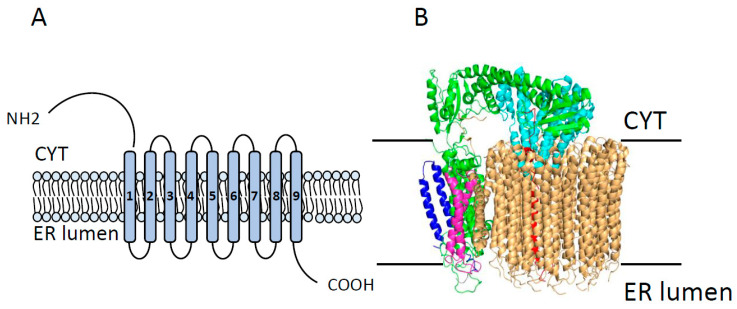
Overall structure of the V0 domain of V-ATPase. (**A**) Topology of V0 domain showing its 9 transmembrane segments with large cytoplasmic N-terminal and C-terminal domains. (**B**) The overall architecture of the V0 domain of V-ATPase (Protein Data Bank [PDB] code 6C6L), showing all known components of the V0 domain, including subunits a (in red), d (in cyan), e (in blue), f (in pink), and the c-ring (in wheat). CYT = cytoplasm; ER lumen = endoplasmic reticulum lumen. Modified from Roh et al. 2018.

Vacuolar ATPase (V-ATPase) is a large multi-subunit P-type ATPase, present in both vacuolar membranes and plasma membranes and which is involved in controlling cellular pH [76]. Structurally, V-ATPase consists of two domains. First is a peripherally associated domain V1 composed of eight (A–H) isoforms which is responsible for ATP hydrolysis. The second subunit is a membrane-associated domain V0 which is made of six different subunits (a, c, c’, c”, d, e) and is responsible for proton translocation (Figure 4) [77,78]. Among these subunits, V0a plays critical roles in membrane distribution and activity range as well as in fine-tuning of the pump [79,80]. Functionally, ATP binding and hydrolysis by the V1 domain are coupled with 360° rotation of the V0 domain and active transport of 2–4 cytosolic H^+^ across the membrane [81,82]. The proper functioning of the V-ATPase is essential for the control of cytosolic, organellar, and extracellular milieu pH which, in turn, is necessary for the appropriate regulation of cellular processes including cell survival and growth [83,84]. Hereby, disturbances in the expression and/or function of V-ATPase have been associated with many diseases, including cancer [85,86,87].

In tumours, the expression level of V-ATPase is often upregulated [83]. For instance, V-ATPase is highly expressed in cervical adenocarcinoma compared to normal tissues and is negatively correlated with patient survival [88]. In gastric cancer, overexpression of the V1A subunit is linked to tumour grade advancement, vascular invasion, and lymph node metastasis as well as reduced patient survival [89]. Similarly, V-ATPase is found upregulated in several aggressive cancers, including breast [90], melanoma [91,92], esophageal [93,94], and pancreatic cancers [95] that further highlights its potential as a prognostic biomarker for advanced metastatic cancers. Based on the subcellular location of the V-ATPase, two mechanisms have been proposed for promoting cancer progression and metastasis in different malignancies. First, in melanoma, breast, and prostate cancers, V-ATPase is located in the plasma membrane where it is responsible for creating an acidic extracellular environment critical for matrix metalloprotease- and protease-mediated cell growth and invasion [96,97,98,99]. Second, in bladder and breast cancers, the vacuolar V-ATPase promotes lysosomal acidification, lysosomal trafficking to the cell surface, and secretion of premetastatic peptides such as cathepsins A and B, leading to tumour metastasis [100,101].

Furthermore, overexpression of V-ATPase has been reported to be closely associated with the development of chemoresistance [102,103]. For example, overexpression of V-ATPase is associated with the development of cisplatin resistance in human epidermoid cancer cells (KB/PC4), human prostate cancer cells (P/CDP5) [104], and cisplatin- and vincristine-resistance in leukemia HL-60 cells [105]. Therefore, treatment with V-ATPase inhibitors restores the chemosensitivity of tumour cells through disrupting the pH gradient between the cytoplasm and lysosomal compartment [106,107,108]. It was shown that treatment with V-ATPase inhibitors omeprazole and esomeprazole restored sensitivity to cisplatin, vinblastine, and fluorouracil (5-FU) in chemoresistant melanoma and colon cancer cells [109]. Furthermore, the application of V-ATPase inhibitor Archazolid induced apoptosis in trastuzumab-resistant breast cancer cells [110]. Together, these findings proved the clinical potential of V-ATPases as both prognostic markers and therapeutic targets for cancer.

## 3. Ion Channels

Ion channels are gated aqueous pores involved in the selective movement of ions across biological membranes. In this type of transport, ions are passively moved down their electrochemical gradient [111]. Depending on the mode of activation, ion channels are classified into two classes, voltage-gated ion channels and ligand-gated ion channels. The voltage-gated ion channels open following changes in the membrane potential while the ligand-gated ion channels open in the presence of extracellular ligands, intracellular second messengers, or chemical factors [112]. Once gates of ion channels are open, ion exchanges across the cellular membranes occur that will result in the redistribution of membrane charges and/or activation of endogenous messengers. Therefore, activation of ion channels triggers various signaling pathways essential for cellular processes ranging from membrane excitability to cell survival [2]. Hereby, alterations in the expression or function of ion channels are associated with multiple human diseases, including cancer. In this regard, many ion channels are considered oncogenic proteins which are often associated with chemoresistance [7,8,113]. Recently, several excellent reviews have described the role of organellar channels in cancer progression and therapy [114,115,116,117]. Therefore, this review will focus on the significance of plasma membrane ion channels in cancer progression and chemoresistance.

### 3.1. Ca^2+^ Channels

Ca^2+^ is a universal second messenger that is vital for the proper functioning of the organism [118]. Thus, its cellular level is always subjected to tight regulations, mainly by the activity of three plasma membrane Ca^2+^ channels, voltage-gated Ca^2+^ channels, Orai-mediated store-operated channels, and transient receptor potential-mediated Ca^2+^ channels. It has been revealed that disruption in the expression or function of these channels is often correlated with carcinogenesis and/or chemoresistance [119,120]. Hence, targeting their expression level or function may serve as an effective strategy to improve cancer treatment. In this section, we will provide examples of the role of Ca^2+^ channels in cancer progression and chemoresistance.

#### 3.1.1. Voltage-Gated Ca^2+^ Channels (VGCC)

VGCCs (Ca_V_) are Ca^2+^ channels that open in response to membrane depolarization. Each Ca_V_ consists of a central a1 subunit and three auxiliary subunits, a2δ, b, and g, in a 1:1:1:1 ratio. In mammals, ten distinct members are grouped into three phylogenetic subfamilies: Ca_V_1 (four different isoforms, Ca_V_1.1–4), Ca_V_2 (three different isoforms, Ca_V_2.1–3), and Ca_V_3 (three different isoforms, Ca_V_3.1–3) [121]. Historically, VGCCs are restricted to exciting cells, however, several Ca_V_ channels are functionally expressed in non-excitable cancer cells [7]. Interestingly, alterations in the expression and/or function of different members of the Ca_V_ subfamilies have been observed in various cancers, suggesting their role in tumour progression, differentiation, and invasion [122,123,124,125]. For instance, Ca_V_1.2 encoded by the human CACNA1C gene is predominantly expressed in oesophageal squamous cell carcinoma and is correlated with tumour cell differentiation [126]. Likewise, Ca_V_1.3 encoded by the human CACNA1D gene is overexpressed in prostate and endometrial cancers [127,128]. Hereby, the Ca_V_1 inhibitor BK10040 was reported to reduce proliferation and induce apoptosis in cancer cells such as A459 (lung adenocarcinoma) and MiaPaCa2 (pancreatic cancer cells) cell lines. Together, these pieces of evidence reveal the oncogenic role of the Ca_V_1 subfamily in human cancers.

As members of the Ca_V_1 subfamily, different Ca_V_2 channels have been reported to be dysregulated in cancer. For example, Ca_V_2.3 encoded by the human CACNA1E gene is upregulated in Wilm’s tumours (a rare childhood kidney cancer), and its expression level is associated with reduced relapse-free survival [129]. Similarly, Ca_V_3.1 and Ca_V_3.2 are highly expressed in human laryngeal carcinoma and glioblastoma, respectively, and their inhibition (using siRNA or mibefradil) causes cell cycle arrest and apoptosis [130,131]. Moreover, the functional expression of Ca_V_ channels has been also shown to be associated with chemoresistance. For example, overexpression of the regulatory subunit α2δ (encoded by human CACN12D3 gene) sensitized esophageal squamous cell carcinoma cell lines to cisplatin-induced cell death. Therefore, genetic silencing of α2δ promoted cisplatin resistance in vitro and in vivo [132]. Likewise, a combination of mibefradil (a blocker of Ca_V_3 subfamily) and carboplatin synergistically inhibited the growth of platinum-resistant ovarian cancer cell lines A2780Cis and IGROV-1, suggesting that a combinatorial drug therapy using Ca_V_ inhibitors and conventional chemotherapeutics may serve as an effective treatment for ovarian cancer [133].

#### 3.1.2. Orai-Mediated Store-Operated Ca^2+^ Entry

Orai proteins are highly selective Ca^2+^ channels that open in response to reduced ER Ca^2+^ levels. Hence, these channels are called store-operated channels (SOC). Currently, three Orai isoforms have been described (Orai1, Orai2, and Orai3), and each of them consists of six subunits that form a single pore [134]. In cancer, the altered expression profile of Orai isoforms is linked to cancer progression [120,135,136,137]. For instance, Orai1 is predominantly upregulated in gastrointestinal stromal tumours and its inhibition (using shRNA or 2-aminoethyl diphenylborate (2-APB) and SKF-96365) decreased proliferation and induced apoptosis in GIST-T1 cells [138]. Similarly, in esophageal squamous cell carcinoma, Orai1 upregulation is correlated with poor overall and recurrence-free survival, therefore its knockdown suppresses tumour growth and metastasis in nude mice xenograft [139]. Like Orai1, upregulated Orai3 has been reported in many cancers. For instance, in breast and non-small lung adenocarcinoma, elevated Orai3 plays essential roles in cell cycle progression, proliferation, apoptosis evasion, and invasion [140,141]. Similar observations have been made in prostate cancer where Orai3 overexpression is positively correlated with aggressive cancer phenotypes and poor clinical prognosis [137].

Furthermore, the expression profile of Orai channels has been associated with chemoresistance in many cancers [142]. For example, Orai1 expression is upregulated in cisplatin-resistant ovary carcinoma cells compared to their parental cells, and its pharmacological inhibition by 2-APB enhances cisplatin-induced cell death in resistant cell lines [143]. In hepatocellular carcinoma, blockade of Orai1 by SiRNA or SKF-96365 enhances cytotoxicity of 5-FU, whereas its ectopic expression induces resistance to 5-FU [144]. Similarly, in pancreatic adenocarcinoma cells, siRNA-mediated silencing of Orai1 enhances 5-FU- and gemcitabine-induced cell death [145]. Moreover, the Orai3 level is upregulated in breast cancer patients and is correlated with poor response to chemotherapy and poor patient outcome. Likewise, Orai3 overexpression in T47D breast cancer cells confers resistance to pro-apoptotic agents (thapsigargin and staurosporine) and chemotherapeutics (cisplatin, 5-FU, and paclitaxel) [146].

#### 3.1.3. TRP-Mediated Ca^2+^ Transport

**Figure 5:**
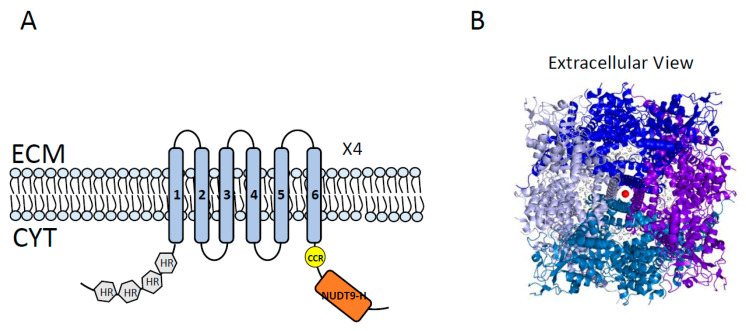
Topology diagram and 3D structure of TRPM2. (**A**) Topology of TRPM2 showing its 6 transmembrane segments (TMS), a channel pore between TMS 5 and 6, and cytoplasmic N- and C-termini. N-terminal harbors 4 TRPM homology domains (shown in gray). C-terminal has a short coiled-coil region (CCR, shown in yellow) followed by the NUDT9H domain (shown in orange) which is the homolog of soluble mitochondrial ADPRase NUDT9. A functional TRPM2 is composed of four homotetramers TRPM2 (x4) and requires binding and hydrolysis of ADP-ribose (ADPR) by NUDT9-H. ECM = extracellular milieu; CYT = cytoplasm. (**B**) Overall domain architecture of TRPM2 (Protein Data Bank [PDB] code 6CO7), showing the extracellular view of four units of TRPM2 surrounding the channel pore (shown in different colors: blue, purple, marine, and light blue, “—” in a clockwise direction).

The transient receptor potential (TRP) family of ion channels consists of 28 distinct members divided into seven subfamilies: TRPC (Canonical), TRPV (Vanilloid), TRPM (Melastatin), TRPML (Mucolipin), TRPP (Polycystin), TRPA (Ankyrin), and TRPN (No mechanoreceptor potential C, nompC) [147]. Structurally, TRP channels show a modular architecture which consists of four concatenated subunits, each composed of six transmembrane segments (TMS) with a channel pore located between TMS 5/6 as well as cytoplasmic N- and C-termini (Figure 5) [148]. Functionally, TRP channels operate as sensory transduction ion channels, which means they sense and translate environmental stimuli into various signal transduction pathways essential for several cellular processes ranging from survival to cell death [149]. TRP channels mediate these effects mainly by changing the intracellular concentration of Ca^2+^, either directly by conducting Ca^2+^ entry or indirectly by changing membrane potential and providing a driving force for Ca^2+^ entry by other channels [150,151]. Therefore, disruptions in the functional expression of TRP channels have been associated with several diseases, including cancer [152,153,154,155,156]. The following sections will focus on the major subfamilies of TRP channels TRPC, TRPV, and TRPM.

##### TRPC

The TRPC subfamily includes seven members (TRPC1–7) which are ubiquitously expressed and play important roles in the regulation of several Ca^2+^-dependent cellular processes [157]. In cancer, TRPCs display diverse functional expressions. For instance, increased expression of TRPC1 in human breast ductal adenocarcinoma samples compared to the adjacent non-tumoural tissues strongly correlates with tumour progression and invasion [158], therefore its silencing suppresses TRPC1-mediated Ca^2+^ entry and reduces cell proliferation [159,160]. Similar observations were established in other cancers including glioblastoma, pancreas, and colon cancers [161]. Moreover, TRPC3 is overexpressed in human ovarian cancer tissues, and its blockade decreases in vitro and in vivo growth of ovarian cancer cells [162]. Further studies show that TRPC3 has a predominant role in the proliferation and migration of a variety of tumour cells, including melanoma, lung, and bladder carcinoma cell lines [163]. Similarly, TRPC6 has been reported to be upregulated in various cancers, including glioma [164], gastric cancer [165], and breast cancer [166], whereby its silencing reduced growth and migration of cultured cells as well as tumour formation and metastasis in nude mice xenografts. Together, these findings highlight that the increased expression levels of TRPCs 1, 3, and 6 are strongly associated with malignant phenotypes of human cancers. On the contrary, TRPC4 is markedly downregulated in renal cell carcinoma cell lines and is correlated with tumour angiogenesis [167]. Therefore, pharmacological activation of TRPC4 by englerin A inhibits growth of A-498 and A-673 cells, suggesting that TRPC4 plays a tumour suppressor activity in renal cancer [168].

Moreover, the altered expression profile of various members of the TRPC subfamily has been associated with chemoresistance. For instance, TRPC1 expression is significantly decreased in cisplatin-resistant (A2780 and SKOV3) and carboplatin-resistant (A2780) ovarian cancer cell lines, suggesting that the reduced expression of TRPC1 is linked to chemoresistance [169]. On the contrary, TRPC5 has increased in 5-FU-resistant colorectal cancer cells HCT-8/5-FU and LoVo/5-FU cell lines, therefore blockade of TRPC5 promotes chemosensitivity in these cells [170]. Similarly, TRPC5 expression was induced following doxorubicin treatment in MCF-7, T47D, and MDA-MB-231 breast cancer cells, and its inhibition restored the cytotoxic effects of doxorubicin [171]. Furthermore, elevated TRPC5 in circulating exosomes negatively correlates with chemotherapy outcome in colorectal and breast cancer patients [172], suggesting that increased TRPC5 is associated with chemoresistance. Similarly, TRPC6 was induced by doxorubicin treatment in Huh7 and HepG2 hepatocellular carcinoma cells, therefore its inhibition enhanced doxorubicin-induced cell death [173], suggesting that high TRPC6 level is associated with chemoresistance in hepatocarcinoma cell lines.

##### TRPM

The TRPM subfamily consists of eight members, TRPM1–8, and each member represents different Ca^2+^ permeability, ranging from Ca^2+^- impermeable channels (TRPM4/5, see Na^+^ channels below) to highly Ca^2+^-permeable channels (TRPM6/7) [174]. Altered expression or function of TRPM channels is associated with the etiology of various cancers. For instance, decreased TRPM1 is linked to the aggressiveness of melanoma tumours and poor overall survival of melanoma patients, suggesting a tumour suppressor role for TRPM1 [175,176,177]. In contrast, upregulated TRPM2 is correlated with poor overall survival in patients with neuroblastoma and gastric cancer [178,179]. Furthermore, inhibition of TRPM2 expression or function decreased growth and invasion of various cancer cells, including breast, gastric, pancreatic, prostate, head and neck, melanoma, neuroblastoma, leukemia, and lung cancers [180,181,182]. Similarly, TRPM3 has been found upregulated in clear cell renal cell carcinoma cell lines 786-O or A498 and its knockdown or inhibition suppressed growth of tumours generated from renal carcinoma cells in orthotopic xenograft mouse models, suggesting an oncogenic role for TRPM3 in renal cancer [183]. Like TRPM2, TRPM7 is overexpressed in various malignancies [184]. For example, upregulated TRPM7 in pancreatic [185,186], breast [158], ovarian [187], and bladder [188] cancers is correlated with tumour progression and aggression as well as poor overall survival of cancer patients, suggesting a protumour effect of TRPM7. Similarly, TRPM8 is predominantly overexpressed in breast [158,189], pancreas [190,191], and prostate [192,193] cancers where its expression correlates with increased cancer cell proliferation and invasion as well as reduced apoptosis and poor patient survival. In contrast, TRPM8 activation by menthol (a natural ligand for TRPM8) reduced survival of melanoma cells, suggesting its anticancer role in melanoma [194].

Furthermore, the altered expression of several TRPM channels has been associated with anticancer drug resistance. For instance, inhibition of TRPM2 expression or function increased the cytotoxic effect of paclitaxel and doxorubicin in breast and gastric cancer cells [178,195]. A similar effect was observed in TRPM2-depleted neuroblastoma cell lines following treatment with doxorubicin [179]. On the contrary, TRPM7 is downregulated in doxorubicin-resistant colon cancer cell line LoVo-R, therefore its silencing confers further resistance against doxorubicin, suggesting that the reduced expression of TRPM7 is linked to doxorubicin resistance in these cells [196]. A similar chemoresistance-promoting effect was observed for TRPM8 in several cancers. For example, TRPM8 overexpression induces resistance to paclitaxel in prostate cancer cells [197]. Moreover, TRPM8 knockdown in osteosarcoma cells enhances the cytotoxic effect of epirubicin [198]. These pieces of evidence suggest that elevated TRPM8 promotes chemoresistance in prostate cancer and osteosarcoma cell lines.

##### TRPV

All six members of the TRPV subfamily show variable permeability to Ca^2+^. While TRPV1–V4 are modestly permeable to Ca^2+^, TRPV5 and TRPV6 represent high Ca^2+^ selectivity. In cancer, the expression profile of TRPV channels is highly contextualized; therefore, depending on the cell and tumour type, TRPV channels can act as both tumour promoters and suppressors [199]. For instance, TRPV1 expression is significantly decreased in melanoma tissues and is inversely related to patient survival. Hereby, activation of TRPV1 expression or function inhibits in vitro and in vivo proliferation of melanoma cells [200]. Similar observations have been made in colorectal and renal cancer cells, therefore TRPV1 activation in those cells inhibited proliferation and induced apoptosis [201,202,203]. Furthermore, TRPV1 depletion causes the spontaneous growth of intestinal tumours, highlighting the tumour suppressor function of TRPV1 in intestinal cancer [204]. On the contrary, TRPV1 was found overexpressed in prostate and breast cancers, therefore its inhibition decreased cancer cell survival [205,206,207]. Together, these findings suggest that TRPV1 can act as both a suppressor and an oncogene, based on the biological context.

Furthermore, enhanced expression of TRPV2 in triple-negative breast cancer [208], bladder cancer [209], and esophageal squamous cell carcinoma [210] is linked to cancer progression and poor patient survival. Conversely, reduced TRPV2 expression has been detected in advanced glioma, therefore its exogenous overexpression negatively affected the in vitro and in vivo proliferation of glioma cells, indicating that in different cancers, TRPV2 may function either as a tumour promoter or tumour suppressor [211]. TRPV3 was also found upregulated in colorectal and lung tumours [212,213] and its inhibition caused cell cycle arrest and decreased cancer cell proliferation [212]. Similarly, elevated levels of TRPV4 in breast, gastric, ovarian, and colon cancers correlates with increased cancer cell proliferation, invasion, and poor patient survival [214,215,216]. On the contrary, the expression level of TRPV4 is significantly reduced in advanced endothelial and skin cancers [211,217], suggesting that TRPV4 exhibits both oncogenic or tumour suppressor effects depend on the cancer type [218]. Moreover, TRPV5 was found downregulated in lung [219] and renal [220] tumours, and its reduced expression correlates with poor overall survival and short relapse-free survival of lung cancer patients [219]. On the other hand, upregulated TPPV6 in various cancers, including breast, colon, prostate, parathyroid, and thyroid cancers, enhanced tumour development and progression [221,222]. However, TRPV6 was reported to be downregulated in other cancers such as esophageal [223], lung [219], and renal [220] cancers, indicating a dual function for TRPV6 as a tumour suppressor or tumour promoter in different cancer types.

In addition to the biological roles and prognostic values, TRPVs have been reported to be involved in the regulation of chemoresistance. In this regard, activation of the TRPV1 channel has been shown to enhance the cytotoxic effects of 5-FU [224], cisplatin [225], and doxorubicin [226] in MCF-7 breast cancer cells. Similar effects were observed in bladder cancer cell lines 5637 and T24 after pirarubicin treatment in the presence of activated TRPV1 [227]. Using a molecular dynamic simulation of TRPV1, Ortega-Guerrero et al. demonstrated that TRPV1 channels can mediate doxorubicin diffusion and promote doxorubicin resistance [228], highlighting the therapeutic benefit of TRPV1 activation for improving the efficacy of the conventional chemotherapy drugs. Moreover, overexpression of TRPV2 in MZC glioma cells induces spontaneous chemoresistance [229]. Similarly, TRPV2 activation enhances the cytotoxic effects of temozolomide (TMZ), carmustine (BCNU), and doxorubicin in U87MG and MZC glioma cell lines [230]. TRPV2 activation also promotes bortezomib-induced cell death in RPMI and U166 melanoma-derived cell lines [231], suggesting that combinatorial treatments using TRPV2 activators and chemotherapeutics may represent an effective strategy to improve cancer therapy. In contrast, increased expression of TRPV6 in prostate cancer cell lines LNCaP and PC-3 correlates with resistance to cisplatin and thapsigargin, hence TRPV6 inhibition enhances cytotoxic effect of these drugs [232].

### 3.2. K^+^ Channels

**Figure 6:**
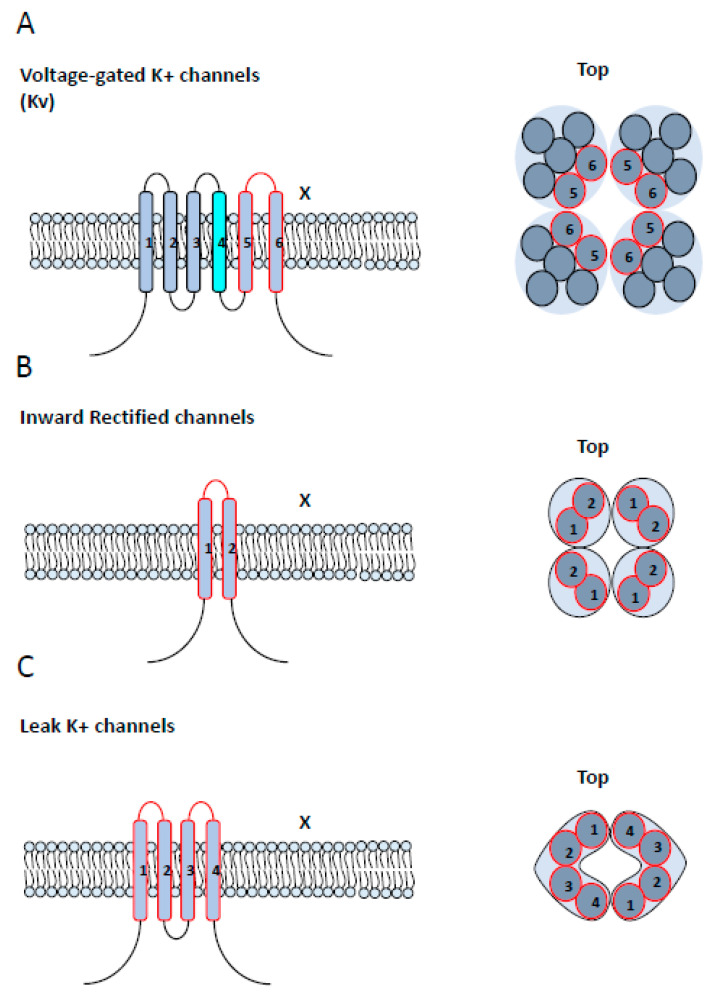
Structural classification of K^+^ channels. (**A**) Left: the voltage-sensitive (Kv) and calcium-sensitive K^+^ (Kca) channels show a similar structure with six transmembrane segments (TMS) and a pore-domain formed by TMS 5 and 6, shown in red. The Kv channels differ in that they contain a voltage sensor in TMS4, shown in cyan. Right: A top view of Kv and Kca channels, showing the six TMS of each of the four subunits and their corresponding pore-forming loops, shown in red. The functional channel is a tetramer protein (x4). (**B**) Left: a lateral view of monomers of an inward rectifier potassium channel (Kir), showing two TMS connected by a pore-forming loop, shown in red. Right: a top view of Kir channel, showing the convergence of four units of Kir channels to the channel pore. The functional channel is a tetramer protein (x4). (**C**) Left: topology of a two-pore domain potassium channel (K2P), showing four TMS and two pore domains. Right: a top view of K2P channel, showing the convergence of two units of K2P to form the channel pore. The functional channel is a dimer protein (x2).

Potassium channels (K^+^ channels) are a diverse and ubiquitous group of ion channels involved in the maintenance and regulation of K^+^ gradients. Given the essential role of K^+^ in the control of cell homeostasis and functions, the proper functioning of K^+^ channels is crucial for a wide array of cellular functions, ranging from membrane excitability to cell proliferation, migration, and apoptosis [233]. Currently, 78 K^+^ channels have been identified and divided into four main classes based on their structural and biophysical characteristics: voltage-gated K^+^ channels (Kv) [234], Ca^2+^- activated K^+^ channels (KCa) [235], inwardly rectifying K^+^ channels (Kir) [236], and two-pore domain K^+^ channels (K2P) [237]. Kv, KCa, and Kir channels have a modular structure which consists of four subunits that contribute equally to the formation of a central tetrameric pore. The only observed structural difference is that each subunit of Kv and KCa consists of six transmembrane segments (TMSs), while Kir subunits possess two TMSs [238]. On the other hand, K2P channels consist of two subunits, each possessing four TMSs harboring two pore domains, which function as a dimer to form a pseudotetrameric pore (Figure 6) [239].

Alterations in the functional expression of K^+^ channels have been associated with the etiology of many cancers [240,241]. For instance, Kv1.1 is markedly upregulated in human medulloblastoma and its knockdown reduces in vitro cell growth and improves survival of tumour-bearing mice [242]. Similarly, KCa3.1 is upregulated in various cancers, including intrahepatic cholangiocarcinoma [243], breast cancer [244], and clear cell renal carcinomas [245], and its expression level correlates with tumour progression and poor patient survival. Likewise, elevated expression of Kir2.1 in advanced gastric cancer is associated with both in vitro and in vivo invasion and metastasis [246]. K2P2.1 is upregulated in prostate cancer and its knockdown induces cell cycle arrest and inhibits cell proliferation [247]. Furthermore, K^+^ channels show differential expression patterns between different cancers and within the same cancer. For instance, Kv11.1 is overexpressed in HT-29 colorectal cancer cells while it was found downregulated in lung carcinoma A549 cells [248]. Likewise, Kv1.3 is upregulated in LNCaP while its expression is reduced in PC3 prostate cancer cells [249].

Moreover, the expression profile of K^+^ channels can be used to predict cancer cell response to anticancer drugs. The expression level of KCa1.1 channel is reduced in cisplatin-resistant ovarian cancer cells and its knockdown further promotes resistance to cisplatin [250]. Similarly, downregulated KCa2.3 is correlated with platinum resistance in ovarian cancer tissues and poor overall survival of ovarian cancer patients [251]. Reduced expression of KCa3.1 is associated with cisplatin-resistant in epidermoid cancer cells, therefore KCa3.1 activation enhances cisplatin-induced apoptosis in these cells [252]. Furthermore, increased Kv1.5 enhances the cytotoxic effects of doxorubicin in gastric cancer cells, hereby its inhibition promotes chemoresistance [253]. This evidence indicates that decreased expression or activity of several K^+^ channels is positively correlated with chemoresistance, therefore K^+^ channel activators can enhance the therapeutic efficacy of conventional chemotherapy drugs. On the contrary, elevated expression of a few K^+^ channels has been shown to limit the efficacy of various chemotherapeutics. Therefore, inhibition of Kv10.1 promotes doxorubicin- and paclitaxel-induced cell death in breast cancer cell lines [254]. Likewise, Kv11.1 inhibition was reported to enhance the cytotoxic effects of cisplatin in colorectal cancer cells [255]. Together, these findings suggest that activation or inhibition of K^+^ channels may represent an effective therapeutic approach for improving cancer treatment.

### 3.3. Na^+^ Channels

Sodium channels (Na^+^ channels) are crucial for membrane excitability and cell communication. Depending on their mode of activation, two distinct classes of Na^+^ channels have been described, voltage-gated sodium channels (VGSC or Na_V_ channels) which open in response to changes in membrane voltage, and ligand-gated sodium channels (LGSC or Na_L_ channels) which are activated by the binding of specific ligands. The following sections focus on the significance of Na^+^ channels in cancer progression and their impacts on chemoresistance.

#### 3.3.1. VGSCs (Na_V_ Channels)

**Figure 7:**
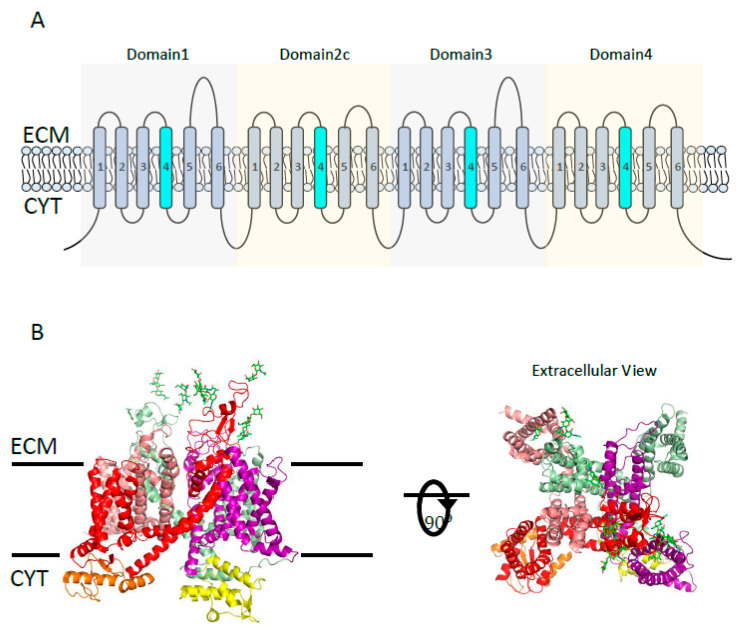
NaV channel structure. (**A**) The topology of the α subunit of NaV channel, showing 24 transmembrane segments (TMS) and four domains (D1–4). Each domain consists of 6 TMS, a pore between the 5/6 TMS, and TMS4 as a voltage sensor (shown in cyan). (**B**) Overall 3D-architecture of the α-subunit of eukaryotic NaV channel (Protein Data Bank [PDB] code 5XOM). Left: the side view of the ΝaV channel domains D1 (red), D2 (salman), D3 (smudge), and D4 (purple) are shown with N- and C-terminal domains colored in orange and yellow, respectively. Glycosylations located in the extracellular loops of D1 and D3 are represented by green sticks. Right: the extracellular view of the NaV channel showing four domains surrounding the channel pore. ECM = extracellular milieu; CYT = cytoplasm.

Na_V_ channels consist of one pore-forming α1 subunit and one or more regulatory β subunits. There are nine different α1 subunits, Na_V_1.1 to Na_V_1.9, which all show a modular structure consisting of four domains (I–IV), each of which contains six transmembrane segments (TMSs) (Figure 7). The four domains form a pseudotetramer around a central pore. One or two out of four β subunits (β1–β4) can associate with α1 subunits to regulate biophysical properties and membrane stability of the channel [256]. Various combinations of α1 and β subunits generate nine functionally distinct Na_V_ channels which are variably expressed across human cells and tissues [257]. In addition to the common role of Na_V_ channels in excitable cells, the functional expression of Na_V_ in various non-excitable cells contributes to the regulation of cell functions such as cell proliferation, invasion, and apoptosis [258].

In cancer, Na_V_ expression is often upregulated, therefore inhibitors of Na_V_ channels have been shown to decrease cancer cell invasion [259]. For example, Na_V_1.1 and Na_V_1.3 are highly expressed in ovarian cancer cells, while Na_V_1.2 and Na_V_1.4 are predominantly overexpressed in highly metastatic ovarian cancer cells compared to low-metastatic cells [260]. Furthermore, expression of Na_V_1.5 is increased in several cancers, including ovarian [260], colon [261], and breast cancers [262], therefore its inhibition significantly impairs in vitro and in vivo invasion of breast cancer cells [262]. Similarly, upregulated Na_V_1.6 was observed in primary cervical cancer cells, and its blockade inhibited invasion of those cells [263]. Furthermore, elevated expression of Na_V_1.7 induces growth and invasion of prostate [264], gastric [265], and endometrial [266] cancer cells. Like α1 subunits, the expression level of non-pore-forming β subunits of Na_V_ channels is altered in various cancers. For instance, the expression level of the β1 subunit is negatively correlated with breast cancer cell migration, hence its overexpression promotes cell adhesion and reduces migration of MDA-MB-231 cells [267]. On the contrary, the β1 subunit was upregulated in breast cancer specimens compared to non-cancer tissues, where its overexpression promoted breast tumour growth and metastasis to the liver and lungs. This evidence suggests that the expression level of β1 subunits can differentially affect cancer progression depending on the tumour cells and tumour microenvironment [268]. A similar controversy has been observed for the β2 subunit, more particularly, overexpression of the β2 subunit induces migration and invasion of LNCaP prostate cancer cells while it inhibits in vivo tumour formation and reduces tumour volume [269]. This example further emphasizes the context-specific effects of β subunits in cancer. Furthermore, expression of β4 subunit is markedly decreased in thyroid [270], breast [271], and cervical [272] cancers, and its overexpression promotes growth and metastasis in MDA-MB-231 breast cancer cells, suggesting that β4 plays a tumour suppressor role in these cancers [271].

Furthermore, the functional expression of Na_V_ channels has been also indicated to be associated with the chemosensitivity of cancer cells [273]. For instance, Na_V_ inhibitor lidocaine enhances the inhibitory effects of cisplatin on breast tumour metastasis and suppresses in vivo formation of lung colonies [274,275]. Lidocaine also enhanced the cytotoxic effects of cisplatin in hepatocellular carcinoma while inhibiting both tumour growth and metastasis [276]. On the contrary, Tran et al. reported that increased expression of Na_V_ channels sensitizes breast cancer to taxol [277]. Likewise, Adashi et al. showed that the expression level of the β3 subunit increased in colon cancer cells following doxorubicin treatment. Together, this evidence suggests that Na_V_ channels can differentially alter cell responses to anticancer drugs [278].

#### 3.3.2. LGSCs (Na_L_ Channels)

Na_L_ channels are activated by the binding of specific ligands. H^+^-Na_L_ and Ca^2+^-Na_L_ channels are the two well-known examples of Na_L_ channels. H^+^-Na_L_ channels are a group of voltage-insensitive Na^+^ channels called acid-sensing ion channels (ASICs). Currently, eight subunits of ASICs have been described: ASIC1a, ASIC1b1, ASIC1b2, ASIC2a, ASIC2b, ASIC3, ASIC4, and ASIC5. Each subunit consists of two transmembrane segments that assemble into homo or heterotrimeric complexes around a central pore [279]. ASICs are widely expressed in human cells and tissues, and their expression has been reported to be associated with the etiology of various cancers. For example, ASIC1 and ASIC3 are functionally expressed in the plasma membrane of lung cancer cells, where they contribute to the acidosis-induced cell proliferation and migration [280]. Upregulated ASIC1 and ASIC3 in prostate cancer cells promote in vitro migration and in vivo tumour metastasis [281]. Furthermore, expression levels of ASIC1 and ASIC2 correlate with the progression of low-grade gliomas to high-grade glioma; therefore, their inhibition decreases in vitro migration of glioma cells [282]. Increased ASIC1 has been also reported in glioblastoma, where its inhibition decreases cell migration [283]. In breast cancer cells, ASIC1 knockdown inhibits in vivo tumour growth and metastasis [284]. Together, this evidence suggests that ASIC channels function as tumour promoters in different cancers.

Furthermore, ASIC1a is highly expressed in 5-FU- and doxorubicin-resistant hepatocellular cancer cell lines (Bel7402/FU and HepG2/DOXO) compared to their parental cells (Bel7402 and HepG2). Hereby, inhibition of ASIC1a by amiloride sensitizes Bel7402/FU and HepG2/DOXO cells to 5-FU and doxorubicin, respectively. Furthermore, exogenous overexpression of ASIC1a in Bel7402 and HepG2 cell lines promotes resistance to 5-FU and doxorubicin. These findings suggest that inhibitors of ASIC channels may serve as potential anticancer drugs by improving chemotherapy [285].

Two common Ca^2+^-Na_L_ channels are TRPM4 and TRPM5, which are monovalent-selective ion channels highly permeable to Na^+^. These channels open in response to increased intracellular Ca^2+^ levels. The altered expression profile of TRPM4 and TRPM5 has been associated with the etiology of several cancers. For instance, TRPM4 is increased in cervical and prostate cancers, and its downregulation reduces cancer cell proliferation and migration [286,287,288]. Similarly, upregulated TRPM5 in several cancers including gastric cancer is linked to poor patient survival. Furthermore, in highly metastatic melanoma cancer cells, TRPM5 expression promotes spontaneous lung metastasis [289]. Together, these studies provide evidence on the tumourigenic effect of Ca^2+^-Na_L_ channels in human cancers.

## 4. Conclusions

Despite advancements in cancer therapy, cancer remains the second leading cause of death worldwide. Nevertheless, over the last few decades, targeted therapy has played a substantial role in improving the overall survival of cancer patients. Since the late 1990s, several small molecules and antibodies raised against specific tumourigenic proteins have been developed and approved by the U.S. Food and Drug Administration (FDA). However, the lack of druggable targets, compounded by severe toxicity profiles, has imposed a significant roadblock for anticancer drug discovery. Hereby, a growing number of studies are being conducted in order to discover novel and effective therapeutic targets for different malignancies. Recently, profound evidence has elucidated that proteins embedded in mammalian plasma membranes may unlock the fundamental basis for understanding carcinogenesis and disarming chemoresistance. Importantly, most ion channels and pumps are located in the plasma membrane and may serve as accessible and druggable targets for cancer treatment. In this regard, several studies have revealed a direct link between the functional dysregulation of membrane transporters and cancer development. Thus, many membrane transporters have been established as potential therapeutic candidates for cancer treatment. A growing body of evidence suggests that modulation of the expression and function of membrane transport proteins not only impacts cancer progression but also alters the cytotoxic effects of chemotherapeutics in different cancers. Given the diverse mechanisms of chemoresistance operating in human malignancies, discovering new therapeutic targets to enhance the efficacy of chemotherapy is crucial for improving patient outcomes. In this review, we emphasized the strong correlation between membrane transport proteins and carcinogenesis by focusing on three main aspects (Figure 3). First, the expression profile of membrane transporters is often altered in cancers, suggesting that membrane transporters may serve as valuable prognostic and diagnostic markers that can be clinically used to improve cancer detection and to monitor cancer progression. Second, the expression and functional patterns of membrane transporters correlate with response to chemotherapy and patient prognosis. Therefore, drugs that can modulate the expression or/and activity of membrane transporters may hold anticancer therapeutic potential, alone or in combination with conventional chemotherapeutics. Third, the strategic location of membrane transporters makes them easily accessible to pharmacological interventions.

Here we presented evidence on the impact of dysregulated membrane transporters on cancer growth, apoptosis, migration, and response to chemotherapy drugs. However, the extensive studies on the biological roles of membrane transporters in cancer are contrasted by a massive lack of information about their intrinsic properties and structural diversity. Hereby, recognizing the intrinsic regulation, gating kinetics, and structural diversity of membrane transporters is a key step toward uncovering their fundamental impacts on cancer progression and chemoresistance. Furthermore, the therapeutic approaches used to target different transporters have been discussed here; however, the lack of specific and potent drugs that can target distinct membrane transporters limits the therapeutic potential of these proteins. More importantly, developing novel drugs targeting membrane transporters requires further understanding of the biological function and structure of these proteins. Therefore, a better understanding of their structure and function may provide greater insights into their role in cancer progression and treatment as well as pave the way for the development of novel anticancer drugs and improvement of current chemotherapy efficacy.

## Appendix A

**Table A1:** Summary of membrane transporter proteins in various cancers, their expression profile, and their role in chemotherapeutic resistance.

Membrane Transport Protein	Type of Cancer	Cell Lines	Level of Expression or Activity	Chemoresistance
Na+/K+ ATPase	Ovarian	2000, CB10	Low	Cisplatin [39], oxaliplatin [45]
	Leukemia	NIH/3T3		Cisplatin [40]
	Lung	PC-14, SBC-1		Cisplatin [41,42]
	Prostate	LNCaP		Cisplatin [43]
	Squamous	BHY		Cisplatin [45]
SERCA	Ovarian	MDAH-2774	Low	Cisplatin [67]
	Lung	H1339		Cisplatin [68]
V-ATPase	Epidermoid	KB/PC4	Hight	Cisplatin [104]
	Prostate	P/CDP5		Cisplatin [104]
	Leukemia	HL-60		Cisplatin, vincristine [105]
	Melanoma	Mel P, Mel M		Cisplatin, vinblastine, & 5-FU [109]
	Colon	Colo1, Colo2		
	Breast	SKRB3, JIMT-1		Trastuzumab [110]
Ca_V_3	Ovarian	A2780	Hight	Carboplatin [133]
	Prostate	LNCaP, PC3		Thapsigargin, Paclitaxel [290]
Orai1	Ovarian	A2780	Hight	Cisplatin [143]
	Hepatocellular	HepG2		5-FU [144]
	Pancreas	Panc1		5-FU, gemcitabine [145]
Orai3	Breast cancer	T47D	Hight	Cisplatin, 5-FU, paclitaxel [146]
TRPC1	Ovarian	A2780, SKOV3	Low	Cisplatin, Carboplatin [169]
TRPC5	Colorectal	HCT-8, LoVo	High	5-FU [170]
	Breast	MCF7, T47D, MDA231		Doxorubicin [171]
TRPC6	Hepatocellular	Huh7 and HepG2	High	Doxorubicin [173]
TRPM2	Breast	MCF7, MDA231	High	Doxorubicin, paclitaxel [195]
	Neuroblastoma	SH-SY5Y		Doxorubicin [179]
	Gastric	AGS, MKN45		Doxorubicin, paclitaxel [178]
TRPM7	Colon	LoVo	Low	Doxorubicin [196]
TRPM8	Prostate	LNCaP	High	Paclitaxel [197]
	Bone	MG-63, U2OS		Epirubicin [198]
TRPV1	Breast	MCF7	High	Cisplatin [225], Doxorubicin [226], 5-FU [224]
	Bladder	5637, T24		Pirarubicin [227]
TRPV2	Glioma	U87MG, MZC	Low	Carmustine, temozolomide, doxorubicin [230]
	Melanoma	RPMI and U166		Bortezomib [231]
TRPV6	Prostate	LNCaP	High	Cisplatin, thapsigargin [232]
KCa1.1	Ovarian	A2780	Low	Cisplatin [250]
KCa3.1	Epidermoid	KB, KCP-4	Low	Cisplatin [252]
Kv1.5	Gastric	SGC7901	Low	Doxorubicin [253]
Kv10.1	Breast	MDA-MB-435S	High	Doxorubicin, paclitaxel [254]
Kv11.1	Colorectal	HCT116	High	Cisplatin [255]
Na_V_	Breast cancer	MCF7, MDA231, MDA468, 4T1	High	Cisplatin [274,275], Taxol [277]
	Hepatocellular	HepG2		Cisplatin [276]
H^+^-NaL – ASIC1a	Hepatocellular	BEL-7402, HepG2	High	Doxorubicin, 5-FU [285]

The switch between the green and orange colors indicates a change in the type of membrane transport protein.
